# Of decrements and disorders: assessing impairments in neurodevelopment in prospective studies of environmental toxicant exposures

**DOI:** 10.1186/1476-069X-14-8

**Published:** 2015-01-21

**Authors:** Sharon K Sagiv, Amy E Kalkbrenner, David C Bellinger

**Affiliations:** Division of Epidemiology, School of Public Health, University of California, 1995 University Avenue, Suite 265, Berkeley, CA California; Department of Environmental Health, Boston University School of Public Health, Boston, MA USA; Zilber School of Public Health, University of Wisconsin-Milwaukee, Milwaukee, WI USA; Children’s Hospital, Harvard Medical School, Boston, MA USA; Department of Environmental Health, Harvard School of Public Health, Boston, MA USA

**Keywords:** Prospective studies, Environmental factors, Neurodevelopmental disorders, Autism spectrum disorders

## Abstract

Prenatal and early life neurodevelopment is exquisitely sensitive to insult from environmental exposures. Identifying the effects of environmental toxicants on neurodevelopmental disorders is particularly important from a public health perspective because many of these exposures are modifiable and may be targeted for intervention. Studying these associations in prospective cohort studies that measure quantitative, dimensional traits related to neurodevelopmental disorders, using standardized instruments such as psychometric tests or rating scales, mitigates many of the challenges that arise when studying clinically diagnosed disorders. We consider validity and feasibility impacts resulting from this design approach, including: 1) enhanced prospective exposure assessment with high quality environmental measures during developmentally relevant windows; 2) reduced bias because studies of continuous outcomes do not recruit cases and controls and are therefore not vulnerable to control selection bias; 3) enhanced statistical power because traits are measured on all individuals in the cohort and power is not limited by the number of cases; 4) reduced outcome misclassification because measuring quantitative traits avoids lumping together individuals with very heterogeneous phenotypes into one category. We use autism spectrum disorders (ASD) as an example to illustrate the advantages of this approach. Investigating the determinants of neurodevelopmental disorders – particularly modifiable determinants such as environmental toxicant exposures – is of great public health importance, given the apparent substantial rise of disorders like ASD over the past few decades. The use of prospective designs measuring quantitative, dimensional traits offers a powerful opportunity to provide important clues to the etiology of these disorders and is likely to accelerate our understanding of the role of environmental toxicant exposures as risk factors.

## Background

The human brain is exquisitely sensitive to the impacts of environmental toxicant exposures during development [1] and can serve as the “canary in the coal mine” for such exposures – acting as an early warning sign for harmful health effects. Identifying the effects of environmental toxicants on neurodevelopment is particularly important from a public health perspective because many of these exposures are modifiable and may be targeted for intervention. Yet, previous epidemiologic studies of developmental toxicants address only a fraction of the thousands of chemicals now in our environment [2]. There is an urgent need to accelerate investigation of environmental toxicants and neurodevelopment and to improve methods to enhance study validity and feasibility.

We propose that prospective cohort studies (often referred to as longitudinal studies when they have repeated measures of an outcome) that measure quantitative, dimensional traits related to neurodevelopmental disorders offer an efficient and valid approach for investigating associations with environmental toxicants. We consider several advantages of this design, and use autism spectrum disorders (ASD) -- a complex disorder with core traits that include impairment in social communication and interaction, and restricted, repetitive patterns of behaviors and interests -- as an illustrative example.

### Prospective studies enhance exposure assessment

Studies of developmental disorders, such as ASD, tend to use case–control designs to ensure a sufficient number of cases of these relatively rare outcomes (ASD prevalence in the U.S. is currently 1.5%) [3]. These designs typically reconstruct historical exposures corresponding to the pertinent developmental window – the prenatal and early life periods – which are likely to be the most critical windows for effects of toxicant exposures on neurodevelopment [4, 5]. Retrospective exposure measurement is typically limited to parent recall or linkage to historical data (e.g. air pollutant monitors), and therefore incurs a moderate degree of exposure measurement error; this error would be expected to attenuate measured associations, possibly masking true associations. In addition, for some toxicants valid retrospective exposure assessment may not be available, precluding them from study with these designs. In contrast, prospective studies, which recruit participants before the development of the outcome, can dramatically improve the quality of exposure data by quantifying environmental toxicant exposures during developmentally relevant windows (time points during development when an insult has greatest potential impact on subsequent development), and employing the most accurate, objective, quantitative measures, such as biomarkers or micro-environmental measurements.

### Prospective cohort studies are not vulnerable to control selection bias

Case–control studies also face threats to validity from problems with control group selection. For example, a selection bias may arise when factors distinguish the ASD case group from the underlying population above and beyond having symptoms of ASD, such as race, socioeconomic position, or access to health services. Children diagnosed with ASD have been shown to differ along these dimensions compared to the underlying population [6]. If the sampling of the control group does not account for these factors influencing diagnosis, the control group will fail to represent the exposure distribution in the population that gave rise to the cases and study results may be biased. In contrast, prospective cohort studies of quantitative, dimensional traits related to a disorder avoid the problem of a potentially biased control group entirely. There is no defined case or control group; rather, all children enrolled are assessed for behavioral traits that fall along a continuum.

### Analyzing continuously distributed traits enhances statistical power

A neurodevelopmental disorder can be conceptualized as an extreme impairment along a dimension of an underlying behavioral trait. For example, individuals with ASD have severe impairment in social cognition. Social cognition can be measured in the general population with neuropsychological tests or parent/teacher-rated behavioral scales, and, like intelligence, exhibits a fairly normal distribution, with ASD falling at the extreme end [7, 8]. A notable limitation of prospective cohort studies is that they are typically underpowered to investigate associations with clinically diagnosed neurodevelopmental disorder due to the rarity of these disorders, leading to insufficient sample size. For example, a prospective study of 1000 participants, considered large by environmental epidemiologic standards, would yield approximately 15 cases of ASD over the duration of the study, which does not lend itself to a feasible analysis. To address limitations in sample size, prospective studies commonly measure dimensional traits related to a disorder. This enhances statistical power because traits are measured on all individuals in the cohort and compared across a range of impairment in relation to an environmental toxicant exposure; power is therefore not limited by the number of cases in the cohort.

### Analyzing quantitative, dimensional traits reduces outcome misclassification

For many aspects of health, the critical issue is not whether an individual “has” the disorder, but “how much of it” [9]. Studies of clinically diagnosed disorder, which are often complex disorders with a range of signs, symptoms, and levels of severity, typically lump together individuals with very heterogeneous phenotypes into one category, often due to statistical constraints (a study would require a large number of participants to look at subtypes of a disorder). Heterogeneity of the case definition for these complex disorders is problematic for etiologic research because different etiologic factors may be at play for the various traits that are included in the diagnostic criteria. This has been cited as a major limitation for research on ASD [10] and the research community has responded with an effort to create new dimensional classifications of mental disorders based on pathophysiology through the Research Domain Criteria (RDoC) initiative [11]. Heterogeneity in current categorical diagnosis introduces noise that could lead to underestimation of an exposure-outcome association. Examination of a single trait shared by cases of a disorder (e.g., impaired social cognition in ASD) reduces outcome heterogeneity and may allow for identification of a more specific exposure-related biologic pathway.

By measuring decrements in a behavioral trait instead of a diagnosed disorder, studies are also immune to temporal changes in diagnostic criteria. For example, the definition of ASD in the Diagnostic and Statistical Manual (DSM) of Mental Disorders has undergone substantial changes in recent years, and these changes have resulted in different ASD prevalence estimates across DSM adaptations [12].

An additional challenge resulting from the heterogeneous nature of these developmental disorders is that children with mild or early manifestations of a disorder may not meet diagnostic criteria but share similar behavioral challenges. This again leads to outcome misclassification, where a subset of individuals does not get classified as cases because they narrowly miss the current diagnostic criteria, but exhibit traits that share etiology with cases. This misclassification does not arise with continuously distributed traits because there is no cutoff or case criteria. Instead, these more mildly affected individuals are more appropriately considered along their numeric range between “normal” and “affected”.

## Limitations

We have focused primarily on the benefits of prospective studies of quantitative, dimensional traits for the study of environmental toxicant exposures. Limitations of this approach exist, including that much of the data collected on behavior comes from rating scales, completed by the parent, teacher or self-completed. These data may be considered more subjective than other neuropsychological test data, however they can provide information about function in multiple settings (e.g., home, school) and are fairly inexpensive and easy to administer. Other quantitative measures, such as brain imaging technologies, while not directly measuring behavioral manifestations, may offer promise in providing objective quantitative measurement of aspects of a neurodevelopmental disorder.

Another limitation is that a given trait, such as impaired social cognition, may be shared by other behavioral disorders, such as attention deficit hyperactivity disorder (ADHD). This overlap could pose challenges for inferring associations with clinically diagnosed ASD, though the high comorbidity of such disorders suggests that they may not be completely etiologically distinct.

From a clinical perspective, exposure-related decrements that do not move a child into a clinically diagnosed category may be considered unimportant because function remains “within normal limits”. However, small decrements in function are important from a population perspective, as small shifts in the mean for a population can translate into an increase in the number of cases of clinically diagnosed disorder in that population [13].

We cite a number of advantages of cohort studies over case–control studies for investigating environmental toxicant exposures and neurodevelopment, however a notable limitation of the prospective cohort design is loss to follow-up. In addition to the expense incurred in following up and tracking participants in cohort studies, loss to follow up can result in selection bias if attrition of participants is related to both exposure and outcome.

## Conclusions

Investigating the determinants of neurodevelopment disorders – particularly modifiable determinants such as environmental toxicant exposures – is of great public health importance, given their apparent substantial rise in prevalence over the past few decades. We conclude that the use of prospective designs measuring quantitative, dimensional traits underlying such disorders confers substantial benefits to the feasibility and validity of epidemiologic studies of environmental toxicant exposures. The risk of failing to pursue such studies is great, including lack of developmental relevance in the measures of exposure, limited statistical precision to resolve potential relationships, low return on investment of data already collected, and false negative findings or missed opportunities when study designs have questionable validity. Studies of quantitative, dimensional traits offers a powerful opportunity to provide important clues to the etiology of neurodevelopmental disorders and are likely to accelerate our understanding of the role of environmental toxicant exposures as risk factors for disorders such as ASD.
